# Molecular Evolution and Spatial Transmission of Severe Fever with Thrombocytopenia Syndrome Virus Based on Complete Genome Sequences

**DOI:** 10.1371/journal.pone.0151677

**Published:** 2016-03-21

**Authors:** Jian-Wei Liu, Li Zhao, Li-Mei Luo, Miao-Miao Liu, Yue Sun, Xiang Su, Xue-jie Yu

**Affiliations:** 1 School of Public Health, Shandong University, Jinan, Shandong Province, China; 2 Department of Pathology, University of Texas Medical Branch, Galveston, Texas, United States of America; University of Minnesota College of Veterinary Medicine, UNITED STATES

## Abstract

Severe fever with thrombocytopenia syndrome virus (SFTSV) was a novel tick-borne bunyavirus that caused hemorrhagic fever with a high fatality rate in East Asia. In this study we analyzed the complete genome sequences of 122 SFTSV strains to determine the phylogeny, evolution and reassortment of the virus. We revealed that the evolutionary rate of three genome segments were different, with highest in the S segment and lowest in the L segment. The SFTSV strains were phylogenetically classified into 5 lineages (A, B, C, D and E) with each genome segment. SFTSV strains from China were classified in all 5 lineages, strains from South Korea were classified into 3 lineages (A, D, and E), and all strains from Japan were classified in only linage E. Using the average evolutionary rate of the three genome segments, we found that the extant SFTSV originated 20–87 years ago in the Dabie Mountain area in central China. The viruses were then transmitted to other areas of China, Japan and South Korea. We also found that six SFTSV strains were reassortants. Selection pressure analysis suggested that SFTSV was under purifying selection according to the four genes (RNA-dependent RNA polymerase, glycoprotein, nucleocapsid protein, non-structural protein), and two sites (37, 1033) of glycoproteins were identified as being under strong positive selection. We concluded that SFTSV originated in central China and spread to other places recently and the virus was under purifying selection with high frequency of reassortment.

## Introduction

Severe fever with thrombocytopenia syndrome (SFTS) was an emerging tick-borne hemorrhagic fever with high case fatality (12 to 50%), and had been reported in most parts of China, South Korea and Japan [1–5]. The major clinical symptoms and laboratory abnormalities of SFTS were non-specific including fever, gastrointestinal symptoms, myalgia, regional lymphadenopathy, thrombocytopenia, leucopenia, and elevated serum hepatic enzymes [1–3]. Severe patients would progress to multiple organ failure or death. The causative agent of SFTS was a novel bunyavirus SFTSV, in genus *Phlebovirus* [1, 6]. Heartland virus, Malsoor virus and Hunter Island Group virus were genetically closely related to SFTSV and were isolated from patients in the United States, bats in India and ticks in Australia, respectively [7–9]. SFTSV was detected in *Haemaphysalis longicornis* [1, 10] and was demonstrated to be transmitted by tick transovarially and transstadially [11]. Person to person transmission of SFTS through contact with infected patient’s blood or mucus had been reported in several clusters of nosocomial transmission of SFTS in China [12, 13]. Animal hosts of SFTSV included domestic animals and wild mammals, such as goats, cattle, dogs, chickens, rodents and shrews [14–17].

Like all the phlebovirus, SFTSV had a three-segmented RNA genome [1, 5]. The L segment contained 6,368 nucleotides encoding the RNA-dependent RNA polymerase (RdRp), while the M segment contained 3,378 nucleotides coding for the envelope glycoproteins Gn and Gc, which played important roles in receptor binding and entrance into the cells. The S segment with 1,744 nucleotides had an ambisense arrangement, which encoded the nucleocapsid protein (N) in a viral complementary sense orientation, and a non-structural protein (NSs) in a viral sense orientation that interacts with interferon signaling pathways and played an important role in evading innate immunity [18–20]. The termini of the three segments of SFTSV were untranslated regions (UTRs) as other bunyaviruses, which were short and highly conserved. Indeed, the 5’ and 3’ UTRs of the three segments were extensively complementary, forming approximately a panhandle-like structure [21].

Mutation and recombination/reassortment were the main revolutionary force for viruses, which might increase their virulence and pathogenicity. In segmented-genome viruses, reassortment was an extremely efficient evolutionary force, which made the viruses fit the new environment, even result in a change of host tropism [22]. Reassortment events had been reported in several segmented viruses and they could cause severe diseases, even death. It was reported that reassortment of RNA segment occurred in SFTSV [23–25].

Bayesian analysis method had been utilized widely to infer the origin and transmission of the virus presently, including influenza virus, Peste des Petits Ruminants virus (PPRV), and Ebola virus (EBOV) [26–30]. Phylogenetic analysis demonstrated that SFTSV could be classified into two clades including six genotypes [25, 31]. Bayesian analysis based on the SFTSV single segment suggested that SFTSV likely originated 50–225 years ago [23, 32]. Bayesian phylogeographic analysis of SFTSV sequences in China suggested that the virus likely originated in Huaiyangshan [23]. Only in China, was spatial transmission analyzed [23], but transmission among these countries in East Asia was still unknown. In our study, analysis based on the SFTSV complete genome sequences was performed to determine the phylogeny, evolution, spatial transmission and reassortment of the virus.

## Materials and Methods

### Data collection

We analyzed only SFTSV that had complete genome sequences for three segments in GenBank (http://www.ncbi.nlm.nih.gov/genbank). The information of each SFTSV strain was collected including GenBank number, host, collecting location and collection date. The collection date of the virus was considered as the date the viral strains was isolated, hence, the range from the collection time to the present was the existence time of the viral strains. In total, 122 SFTSV strains with a time span from 2010 to 2014 were collected from GenBank and analyzed in this study. To analyze the whole genome sequences, the L, M and S segments of each strain were concatenated in the order of L-M-S. The concatenated sequence of L segment, M segment, and S segment of each viral strain was used for phylogenetic analysis.

### Phylogenetic Analysis

The L, M, S and concatenated sequences of SFTSV were aligned using the Clustal W in MEGA5.02 software program (http://www.megasoftware.net/). The phylogenetic trees for analyzing molecular evolution were constructed using the maximum likelihood method based on the Kimura 2-parameter model using the MEGA. The confidence of the phylogenetic tree was tested using 1,000 bootstrap replications. The “use all sites” option was chosen for the Gaps/Missing Data Treatment, and the “1st + 2nd + 3rd + Noncoding Sites” was for the codon positions.

### Reassortment Analysis

Prior to reassortment detection, the concatenated complete genome sequences were aligned using the multiple alignment program Clustal X. To prevent potential biases during phylogenetic inference, the concatenated sequences were analyzed with Recombination Detection Program version 3.44 (RDP3) that incorporates RDP, GENECONV, Bootscan, Maxchi, Chimaera, SiScan and 3Seq methods [33, 34]. Only reassortment events with p-values≦0.05 which were detected by three or more methods were considered, employing the Bonferroni correction to avoid false positive results.

### Selection Analysis

Analysis of selection pressure in individual gene of SFTSV was performed by obtaining mean ratios of non-synonymous (dN) to synonymous (dS) substitutions per site (dN/dS). The overall dN/dS ratios of all genes were calculated by using single likelihood ancestor counting (SLAC) in the HyPhy package [35] on the Datamonkey (http://www.datamonkey.org). The positive selection sites were identified by SLAC, fixed effects likelihood (FEL), internal fixed effects likelihood (IFEL), mixed effects model of evolution (MEME) and the fast unbiased Bayesian approximation (FUBAR) [36]. Only sites detected by at least two methods with statistically significant values were considered as being subject to positive selection under the p-value≦0.05 for SLAC, FEL, IFEL and MEME; posterior probability≧0.95 for FUBAR.

### Bayesian Time-Scaled Phylogenetic Analysis

Prior to determine the molecular evolutionary rate of the four data sets, the clock-like behavior of each data set was assessed using a regression of root-to-tip genetic distances inferred from the maximum likelihood (ML) trees against sampling time in the program Path-O-Gen (v1.4, http://tree.bio.ed.ac.uk/software/pathogen/) [37]. To determine the molecular evolutionary rate and estimate the time to the most recent common ancestor (TMRCA) for each of the data sets, Bayesian coalescent phylogenetic analysis was performed using the Bayesian Markov chain Monte Carlo (MCMC) method available in the BEAST package v1.8.0 (http://beast.bio.ed.ac.uk) [27, 32]. The HKY + G nucleotide substitution model and a flexible non-parametric Bayesian skyride coalescent model were used. To allow for rate variation among lineages, an uncorrelated lognormal relaxed molecular clock was used in all cases. We performed the independent runs for 10^5^ generations, sampling every 2,000 steps. In addition, to accurately estimate the substitution rate, we repeated the analysis. The nucleotide substitution rate (substitutions/site/year) and the TMRCA (year) values were obtained from Tracer v1.5 (http://tree.bio.ed.ac.uk/software/tracer/). Maximum clade credibility (MCC) phylogenetic tree was summarized by using TreeAnnotator (http://beast.bio.ed.ac.uk/treeannotator) and exclusion of the first 10% of the trees as burn-in. The MCC tree with median node heights was visualized in FigTree software v1.4.0 (http://tree.bio.ed.ac.uk/software/figtree/). To test the strength of temporal signal in the data, which is essential to the estimation of substitution rates, we repeated the BEAST analysis (using identical parameters) on a data set in which sampling times were randomized on the tips [37, 38]. The 95% highest posterior densities (HPDs) of these randomized sequences were then compared with those of the real data. If these sequences contain clear temporal structure, then the real and randomized data would have different mean estimated substitution rates, and different distributions. Here, the null distribution of mean substitution rates for each data set was generated 5 times per alignment.

## Results

### Phylogenetic analysis

**Fig 1:**
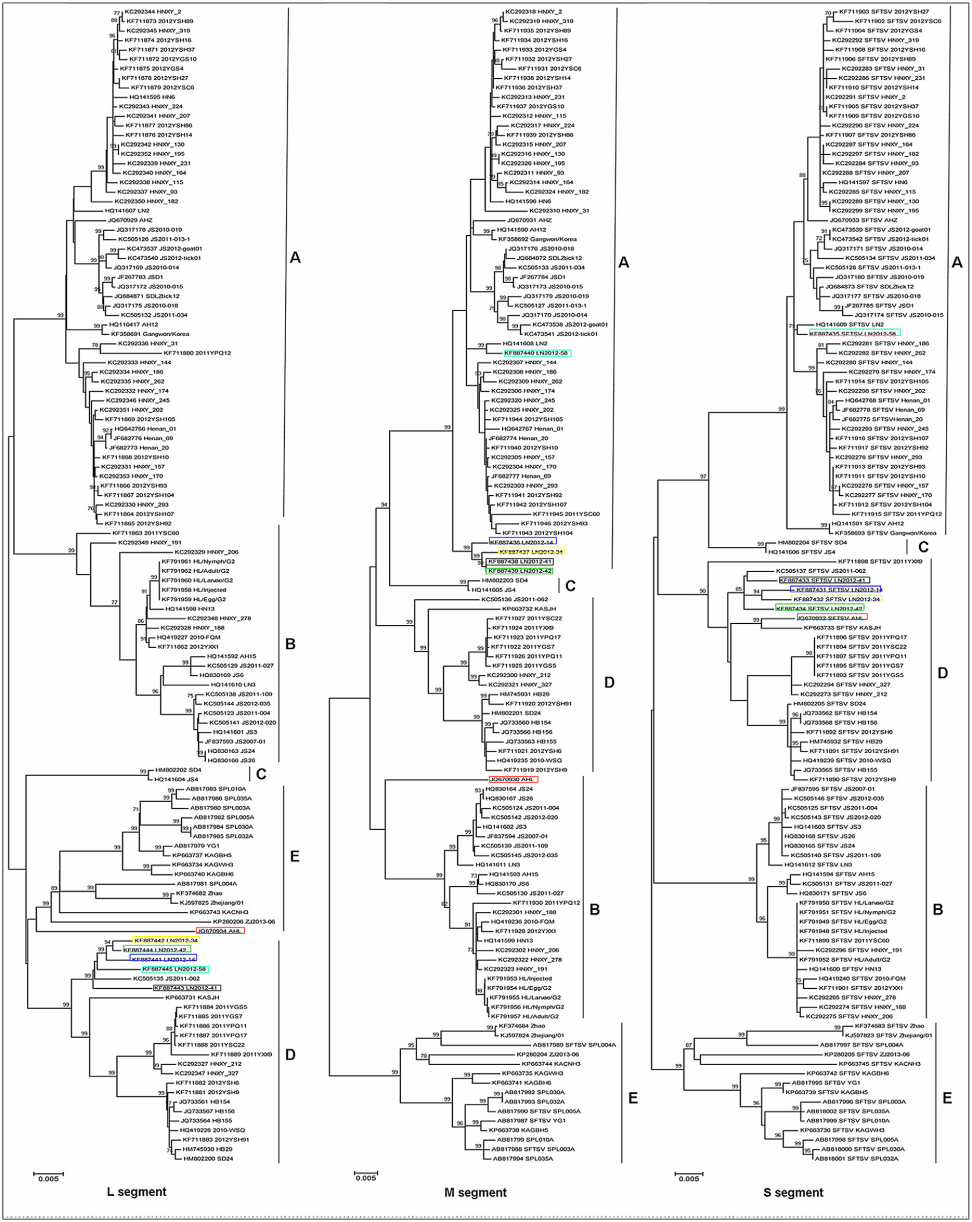
Phylogenetic analysis of the whole segment sequences of L, M, and S segments of 122 SFTSV strains. The maximum likelihood trees were constructed by using MEGA 5.02 software (http://www.megasoftware.net/). SFTSV was classified into 5 lineages labeled as A, B, C, D, and E by each genome segment. GenBank accession number and strain name were labeled on each branch. Bootstrap values ≧70 were labeled at nodes. Scale bar represented nucleotide substitutions per site.

Only SFTSV strains with complete genome sequences were analyzed in this study. Among the 122 SFTSV strains selected, 108 strains were from China. Chinese SFTSV strains were 61 from Henan, 21 from Jiangsu, 8 from Shandong, 7 from Liaoning, 4 from Hubei, 4 from Anhui, and 3 from Zhejiang. Eight SFTSV strains were from Japan and 6 SFTSV strains were from South Korea. All SFTSV strains were isolated between 2010 and 2014. Phylogenetic analysis classified all SFTSV strains into 5 lineages in each segment, named A, B, C, D and E (Fig 1). Lineage A contained the most viral strains (55 strains) and all strains in lineage A came from China (Henan, Jiangsu, Shandong, Anhui, Liaoning provinces) except for 1 strain (Gangwon/Korea) from South Korea. All SFTSV strains in lineage B were from China, including Henan, Jiangsu, Shandong, Anhui, Liaoning provinces. Lineage C included only 2 strains all from China, with 1 strain from Shandong Province, and the other from Jiangsu Province, while most SFTSV in lineage D were from China (Henan, Hubei, Shandong, Anhui, Liaoning provinces) with 1 strain (KASJH) from South Korea. Interestingly, all the 8 strains from Japan, 3 strains from Zhoushan Archipelago in the East China Sea of Zhejiang Province of China, and 4 strains from Jeju Island of South Korea belonged to lineage E. We also found that different segments of the same strain belonged to different lineage, suggesting segment reassortment, e.g. AHL belonged to lineage E in segment L, but it belonged to lineage B and D in segment M and S, respectively.

### Reassortment among SFTSV strains

The concatenated sequences were analyzed by RDP4, suggesting 14 reassortment events in SFTSV complete genomes. Reassortment events existed in M and S segments in eight viral strains: HL/Injected, HL/Egg/G2, HL/Larvae/G2, HL/Nymph/G2, HL/Adult/G2, HN13, 2012YXX1, HN_278. These findings were confirmed by RDP, GENECONV, Bootscan, Maxchi, Chimaera, SiScan and 3Seq and supported by significant p-values of 1.73E-11, 2.35E-8, 3.76E-10, 1.41E-12, 1.72E-9, 5.44E-34, 7.52E-46 respectively. Likewise, the viral strain LN2012-58 was also considered as a reassortant virus in M and S segment because p-values of RDP, GENECONV, Bootscan, Maxchi, Chimaera, SiScan and 3Seq were 2.29E-5, 3.30E-18, 8.45E-6, 1.25E-6, 7.81E-4, 4.22E-14 and 4.07E-5 respectively. Four strains (LN2012-14, LN2012-34, LN2012-41 and LN2012-42) were considered as reassortment events in S segment. These results were found by RDP, GENECONV, Bootscan, SiScan and 3Seq and supported by significant p-values of 1.82E-11, 6.14E-7, 2.18E-11, 1.11E-11, and 1.66E-21, respectively. Besides, strain AHL was a reassortant virus in S segment, with significant p-values of 4.28E-7, 8.40E-8, 9.50E-13, 1.04E-5, 5.10E-4 and 2.41E-12 for RDP, GENECONV, Bootscan, Maxchi, Chimaera and SiScan methods.

Combined with the phylogenetic analysis, secondary screening was performed from the reassortment events analyzed by RDP4. Six strains were identified as reassortment events, including strains LN2012-14, LN2012-34, LN2012-41, LN2012-42, LN2012-58 and AHL. Four SFTSV strains (LN2012-14, LN2012-34, LN2012-41 and LN2012-42) were classified into lineage D in segment L and S, but they belonged to lineage A in segment M. Strain LN2012-58 belonged to lineage D in segment L, while it belonged to lineage A in segment M and S. Strain AHL belonged to lineage E in segment L, but it belonged to lineage B and D in segment M and S (Fig 1).

### Selection Analysis

To assess the selection pressures acting on RdRp, glycoprotein (GP), N and NSs genes, the average dN/dS value measured by using SLAC on online server Datamonkey was shown in Table 1. The dN/dS values of the four genes ranged from 0.026 to 0.092, which indicated that SFTSV was under purifying selection. The highest dN/dS ratio was observed in the GP gene, followed by the NSs, RdRp, N genes.

**Table 1:** Gene-specific global dN/dS ratios estimated using SLAC method.

Genes	dN/dS	95%HPD
RdRp	0.039	(0.035, 0.045)
GP	0.092	(0.082, 0.104)
N	0.026	(0.016, 0.039)
NSs	0.073	(0.056, 0.093)

Two sites (37, 1033) in glycoprotein were identified as being under strong positive selection with one using two different detective methods (SLAC, MEME), the other using four different detective methods (FEL, IFEL, MEME, FUBAR) (Table 2). Other 7 sites were identified as positive selection with only one method FEL or MEME. No positive selection sites were found in RdRp, N and NSs genes.

**Table 2:** Positive selection sites analysis using SLAC, FEL, IFEL and REL methods.

Methods	SLAC*	FEL*	IFEL*	MEME*	FUBAR^#^
Positive sites	**1033**	**37**, 323	**37**	**37**, 524, 554, 642, 651, 926, 956, **1033**	**37**

Bold numbers denote amino acids sites under strong positive selection.

*: p-value ≦ 0.05;

^#^: posterior probability ≧ 0.95.

### Estimating Evolutionary Rates

A conservative assessment of the degree of clock-like evolution present in a data set is achieved by fitting a regression of the year-of-sampling against the root-to-tip genetic distance of each sample, measured from the maximum likelihood tree. The regression coefficient (R^2^) were 0.2504, 0.2838, 0.2231 and 0.1247 for the data set of concatenated sequence of L-M-S and individual segment sequence of L, M and S, respectively, which strongly supported the presence of molecular clock-like structure.

The average evolutionary rate of each genome segment was different, which was estimated by the Bayesian time-scaled phylogenetic analysis. For the L segment, the mean rate was 4.16E-4 substitutions per site per year (s/s/y) ranging from 8.99E-5 to 9.72E-4 (with 95% highest posterior densities, HPD). For the M segment, the mean rate was 6.76E-4 s/s/y (95%HPD = 3.92E-4 to 1.00E-3 s/s/y). The S segment had a mean rate of 1.09E-3 s/s/y (95%HPD = 5.43E-4 to 1.60E-3 s/s/y). The S segment had a highest evolutionary rate, which was 2.62 times of that of L segment and 1.61 times of that of M segment. To estimate the evolutionary rate of the SFTSV complete genomes, the complete concatenated genomes were performed as described above. The mean rate of the concatenated sequences was estimated to be 6.73E-4 s/s/y (95%HPD = 2.35E-4 to 1.09E-3 s/s/y), which was similar to M segment. A Bayesian time-scaled MCC tree based on the complete concatenated genomes was constructed.

**Fig 2:**
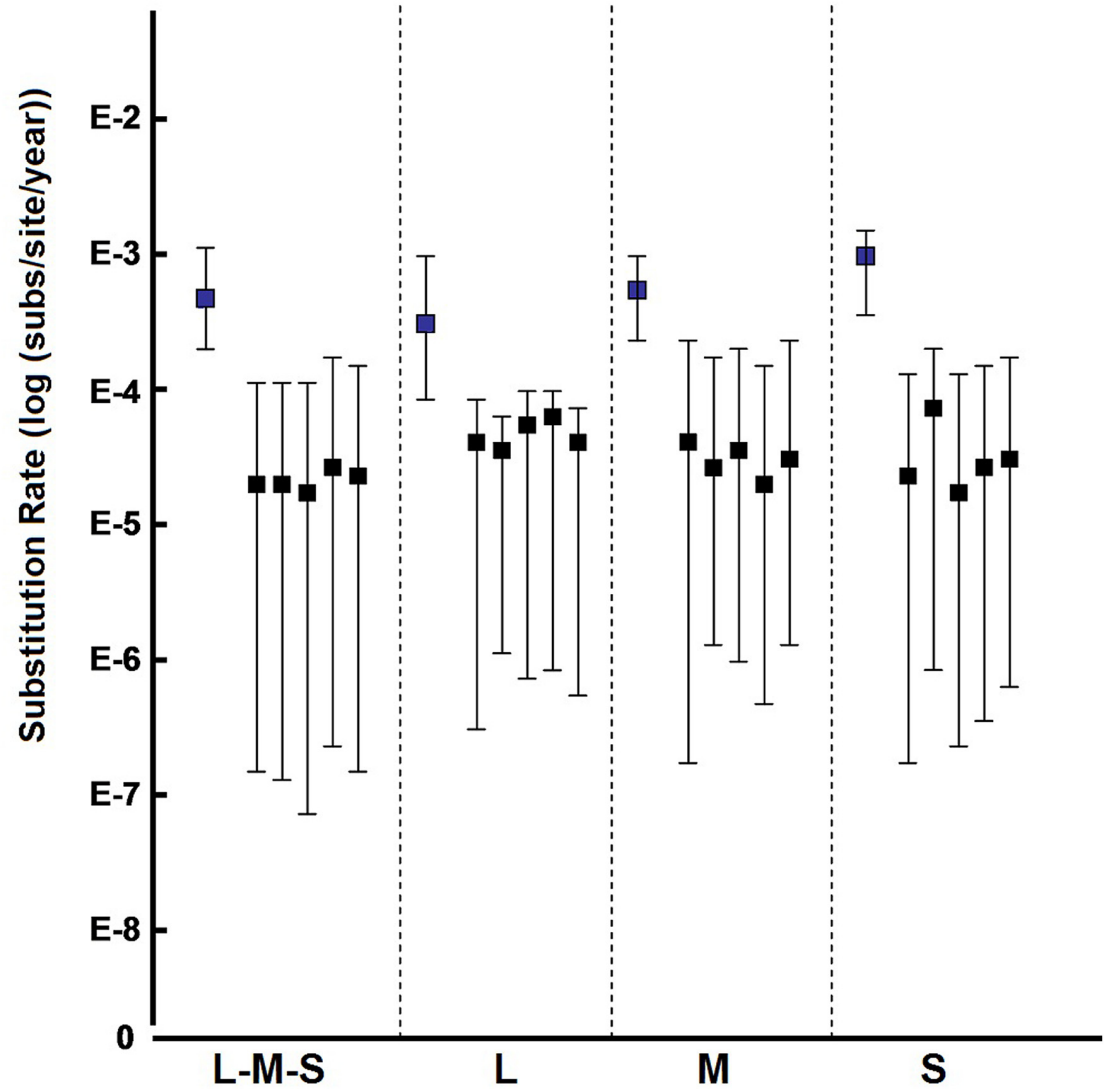
Posterior mean and 95% HPDs of the substitution rates estimated from the actual data sets and the 5 tip-date randomizations for the each data set. Substitution rates on the left for each data set were estimated from the actual data sets. Substitution rates on the right for each data set were estimated from the randomized data sets. The mean rates estimated for the data sets were significantly different from those estimated from the randomized data sets.

Randomized analysis suggested that, the mean evolutionary rates of the actual data did not fall in the 95% confidence intervals estimated from the randomized data sets (Fig 2).

### Temporal and Spatial Dynamic Analysis

**Fig 3:**
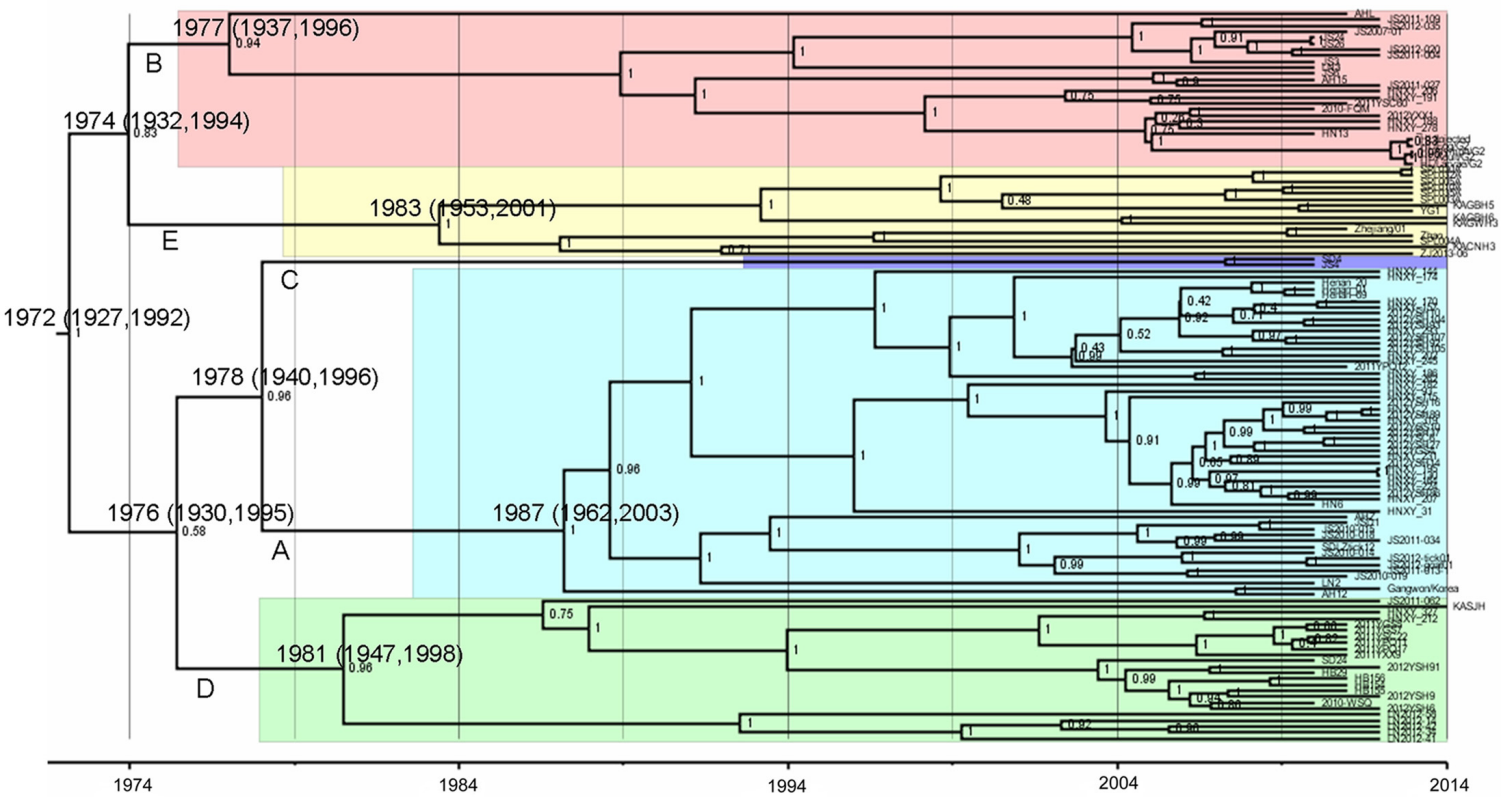
Time-scaled Bayesian MCC phylogenetic tree based on concatenated SFTSV complete genome sequences. Tree nodes were annotated with posterior probability values (right), estimated median dates of time to most recent common ancestor (TMRCA) and 95% confidence interval of TMRCA (above). Lineages (A, B, C, D and E) were marked with different colors. SFTSV strain names were labeled on each branch. Horizontal axis indicated time in years.

All the viral strains were divided into 5 lineages like the L segment described above (Fig 3). The estimated median TMRCA of SFTSV for all 5 lineages were found to be 1972 (95% HPD = 1927 to 1992). The TMRCA were estimated to be 1987 (95% HPD = 1962 to 2003), 1977 (95% HPD = 1937 to 1996), 1981 (95% HPD = 1947 to 1998), 1983 (95% HPD = 1953 to 2001) for A, B, D, E lineages respectively. Lineages A and C diverged from each other in 1978 (95% HPD = 1940 to 1996). In 1976 (95% HPD = 1930 to 1995), lineage D diverged from lineage A and C. The TMRCA were estimated to be 1974 (95% HPD = 1932 to 1994) for lineage B and E.

Temporal and spatial transmission routes were primarily confirmed using the Bayesian analysis based on the 122 complete concatenated genomes from China, Japan and South Korea. The extant SFTSV originated 42 (95% HPD = 20 to 87) years ago in the Dabie Mountain area in central China encompassing Henan, Hubei, Anhui, and Jiangsu provinces. Around 37 (95% HPD = 18 to 77) years ago, the virus moved to Shandong Province from Henan Province, and the virus was transmitted from Jiangsu Province to Liaoning Province in Northeastern China about 33 (95% HPD = 16 to 67) year ago. SFTSV spread to Zhoushan Archipelago of China, Jeju Island of South Korea, and Japan approximately 31 (95% HPD = 13 to 61) year ago, and there was mutual transmission among these areas.

## Discussion

Phylogenetic analysis indicated that all 122 strains in East Asia were divided into five lineages in each segment. In each lineage, there were virus strains from China. SFTSV strains from the mainland of South Korea (Gangwon/Korea and KASJH) were classified into A and D lineages, while four other strains from Jeju Island of South Korea together with SFTSV strains from Zhoushan Archipelago of China and from Japan were classified into lineage E. This was similar to the previous study, which classified SFTSV virus into two clades, Chinese clade and Japanese clade [31]. Bayesian analysis based on the complete concatenated genomes showed the same result, which further explained the close relationships among the viruses from the islands surrounding the East China Sea including the Zhoushan Archipelago of China and Jeju Island of South Korea, and the Japanese Archipelago. The case fatality rate of SFTS patients in Japan was apparently higher than that in South Korea and Zhejiang Province of China [2, 39]. It is not clear the high case fatality in Japan is because of high virulence of the Japanese SFTSV strains or because of small sample size of SFTS cases from Japan especially when retrospective study were used for diagnosis of SFTS, which might be focused on severe or dead SFTSV patients. Japanese SFTSV strains were classified into the Chinese clade from the mainland of China in the previous study [31], but we did not find this phenomenon because the strains had no complete genome sequences and were not analyzed in our study. Strain AHL belonged to lineage E in segment L, but it belonged to lineage B and D in segment M and S, respectively, suggesting that AHL was probably a reassortant. Our subsequent analysis and previous study have identified AHL as a reassortant [23, 24].

Reassortment is distinct from homologous recombination, which is widespread among viruses with segmented genomes, including the *Orthomyxovirus* and *Bunyavirus* [40, 41]. The reassortment event requires that the viruses must have at least two segmented genomes. When two or more segmented viruses co-infected a single cell simultaneously, the genome segments might be packaged into progeny viruses randomly. Then, the progeny might inherit genomic segments from more than one parent, obtaining increased genetic variability because the offspring might contain novel combinations of genomic segments. This process was an important reason for virus survival and diversification, as illustrated by the antigenic shift commonly observed in influenza virus, another negative strand segmented RNA virus [26]. Natural reassortment has been reported for various members of the *Phlebovirus* genus, including *Candiru* virus and Rift Valley fever virus [34, 42, 43]. Reassortment could increase pathogenicity and enhance transmissibility among vectors and hosts [44]. Six SFTSV strains were identified as reassortants in our analysis, which were isolated from human samples, suggesting that reassortment events occurred frequently in SFTSV hosts.

To assess the evolutionary substitution rate, TMRCA, and divergence of SFTSV lineages and the geographic origin of SFTSV, 122 complete genomes were analyzed by the Bayesian Markov chain Monte Carlo (MCMC) method. From a genetic perspective, substitution rates are critical parameters for understanding virus evolution, given that restrictions in genetic variation within a population of viruses can lead to low adaptability and pathogenicity [45]. Our analysis estimated that the evolutionary rate of three segments was different, with 4.16E-4 s/s/y (95%HPD = 8.99E-5 to 9.72E-4 s/s/y) in L segment, 6.76E-4 s/s/y (95%HPD = 3.92E-4 to 1.00E-3 s/s/y) in M segment and 1.09E-3 s/s/y (95%HPD = 5.43E-4 to 1.60E-3 s/s/y) in S segment, which was similar to that predicted for SFTSV in previous studies and other phleboviruses (E-4 s/s/y) [23, 32, 34]. The mean evolutionary rate of the concatenated sequences was estimated to be 6.73E-4 s/s/y (95%HPD = 2.35E-4 to 1.09E-3 s/s/y). Generally speaking, the M segment should have the highest evolutionary rate in bunyaviruses, as it encoded the two glycoproteins which play a role in interaction with receptors in the cell surface. However, higher substitution rates were observed in the S segment because of the variability seen at the nucleotide level, in the highly variable region of the NSs gene sequence for SFTSV.

Temporal and spatial dynamics of RNA viruses are often reflected by their phylogenetic structure [46]. The inference of divergence events presented facilitated a better understanding of historical divergence and offered further opportunities to study viral demographic history and dispersal events [27]. Maximum clade credibility phylogenetic tree supported the TMRCA for all sampled SFTSV strains were found to be 42 (95% HPD = 20 to 87) years ago, and lineage E isolates were predicted to have diverged at the latest time. SFTSV existed earlier than its first description in central China and later in Japan and South Korea. The primary reason was that SFTSV was unknown, and lacked the corresponding detective methods and tools for the new virus. Secondly, changes in the working environment increased contact between the vectors and farmers. Lastly, virulence of the virus increased because of mutations and reassortment events. Our analysis indicated that central China was the geographic origin of the most recent common ancestor of SFTSV because of having the highest viral diversity in the area and viral strains distributing in four lineages sans lineage E. Furthermore, another aspect was the TMRCA from the MCC phylogenetic tree. In conclusion, these findings suggest that SFTSV originated in central China, which then spread to eastern China, northeastern China, and from Zhejiang Province in eastern China it spread to Japan and South Korea. Based on the genotype distribution of SFTSV and Bayesian analysis, Fu et al demonstrated that SFTSV strains in Zhoushan Islands were from central China and South Korea, and the virus in Japan were from South Korea [25]. In Fu’s and our analysis, SFTSV strains in South Korea were classified into three lineages or genotypes. Within the three lineages, one included SFTSV strains only from Zhoushan Islands, South Korea, and Japan, while the other two included the virus from central and northeastern China, and South Korea. Thus, we inferred that SFTSV strains in the mainland of South Korea were transmitted from Liaoning, Shandong or Jiangsu province in China, while SFTSV strains in Jeju Island of South Korea were from Zhoushan Archipelago of Zhejiang Province of China. SFTSV in Japan were transmitted from Zhoushan Archipelago of Zhejiang Province of China or from Jeju Island of South Korea. However, while these predictions are suggestive of a potential origin for SFTSV, cautions must be exercised in their interpretation because limited SFTSV strains from limited areas were used in the analysis. In our study, only 8 strains from Japan and 6 from South Korea were used, which could affect the lineage classification of the phylogenetic analysis and the judgment of the virus origin and transmission routes. To obtain more accurate data of SFTSV lineage classification, origin and transmission routes, analysis should contain more complete genome sequences from South Korea and Japan. Although ticks were considered as a vector of SFTSV, ticks could not fly and with limited activity areas of the mammals, there must be a host to carry ticks from area to area efficiently. We inferred that the host should be migratory birds, but we had no evidence.

SFTSV glycoproteins exhibited the highest dN/dS ratio among the four genes. Theoretically, this might reflect the high antigenicity, which could be under selective pressure to evade the host humoral immune response as well as to adapt to their respective cell-surface receptors for attachment and entry [47]. Although we found two sites in the SFTSV glycoprotein under positive selection, we did not know if a mutation in the two sites could affect the virulence of the virus. Thus further research should be performed based on reverse genetics to identify the relationship between mutation of the sites and the virulence. The NS protein had the highest dN/dS ratio. We found that there were up to 5.2% diversity among all the amino acids of sampled NS genes. The NS protein is thought to affect virulence, primarily through its antagonism of interferon, helping the virus to evade host antiviral responses [18, 19]. The higher dN/dS ratio in NS gene might be the result from a co-evolutionary battle between the virus and host immunity.

## Conclusions

We conclude that SFTSV originated in central China and spread to other places recently and the virus is under purifying selection with high frequency of reassortment.
